# Measuring psychological flexibility in medical students and residents: a psychometric analysis

**DOI:** 10.3402/meo.v18i0.20932

**Published:** 2013-08-13

**Authors:** Christie L. Palladino, Brittany Ange, Deborah S. Richardson, Rhonda Casillas, Matt Decker, Ralph A. Gillies, Amy House, Michael Rollock, William H. Salazar, Jennifer L. Waller, Ronnie Zeidan, Lara Stepleman

**Affiliations:** 1 Communication Research Working Group, Educational Innovation Institute, Medical College of Georgia, Georgia Regents University, Augusta, GA, USA; 2 Department of Obstetrics and Gynecology, Georgia Regents University, Augusta, GA, USA; 3 Office of Evaluation, Medical College of Georgia, Georgia Regents University, Augusta, GA, USA; 4 Department of Psychology and Center for Teaching and Learning, Georgia Regents University, Augusta, GA, USA; 5 Department of Counseling Services, Arizona State University, Tempe, AZ, USA; 6 Department of Psychology and Counseling, Ohio Christian University, Circleville, OH, USA; 7 Department of Family Medicine, Georgia Regents University Augusta, GA, USA; 8 Department of Psychiatry and Health Behavior, Georgia Regents University, Augusta, GA, USA; 9 Department of Medicine, Georgia Regents University, Augusta, GA, USA; 10 Department of Biostatistics, Georgia Regents University, Augusta, GA, USA; 11 Department of Anesthesiology and Critical Care, Hospital of the University of Pennsylvania, Philadelphia, PA, USA; 12 Educational Innovation Institute, Georgia Regents University, Augusta, GA, USA

**Keywords:** psychological flexibility, assessment, mindfulness, psychological well-being, psychometrics, validity

## Abstract

**Purpose:**

Psychological flexibility involves mindful awareness of our thoughts and feelings without allowing them to prohibit acting consistently with our values and may have important implications for patient-centered clinical care. Although psychological flexibility appears quite relevant to the training and development of health care providers, prior research has not evaluated measures of psychological flexibility in medical learners. Therefore, we investigated the validity of our learners’ responses to three measures related to psychological flexibility.

**Methods:**

Fourth-year medical students and residents (*n*=275) completed three measures of overlapping aspects of psychological flexibility: (1) Acceptance and Action Questionnaire-II (AAQ-II); (2) Cognitive Fusion Questionnaire (CFQ); and (3) Mindful Attention and Awareness Questionnaire (MAAS). We evaluated five aspects of construct validity: content, response process, internal structure, relationship with other variables, and consequences.

**Results:**

We found good internal consistency for responses on the AAQ (*α*=0.93), MAAS (*α*=0.92), and CFQ (*α*=0.95). Factor analyses demonstrated a reasonable fit to previously published factor structures. As expected, scores on all three measures were moderately correlated with one another and with a measure of life satisfaction (*p*<0.01).

**Conclusion:**

Our findings provide preliminary evidence supporting validity of the psychological flexibility construct in a medical education sample. As psychological flexibility is a central concept underlying self-awareness, this work may have important implications for clinical training and practice.

## Introduction

Self-awareness regarding one's own feelings, thoughts, and behaviors is a key principle of relationship-centered care (1). However, the notion of one's self-awareness as a provider has been largely understudied in medicine (1, 2). Furthermore, educators are concerned that students in traditional medical curricula may develop self-protective barriers that limit this type of self-reflection (3), and thereby impede the delivery of relationship-centered care. Before we can develop and evaluate interventions that would positively impact learners’ self-awareness, it is important to develop and refine methods for assessing self-awareness and other relevant abilities in this population.

A psychological concept central to the notion of self-awareness is psychological flexibility. The construct of psychological flexibility involves mindful awareness of one's thoughts and feelings but adds the condition that such awareness does not produce barriers to acting consistently with one's values. For example, a physician may find him/herself with negative thoughts and feelings toward obese individuals. The psychologically flexible physician should be more able to acknowledge his/her negative response but still collaborate with the patient in an effective and compassionate way. The less psychologically flexible physician, however, would be more likely to either avoid acknowledging the negative response, which could lead to limiting contact with the patient, or act on the negative thoughts and feelings, which could lead to interacting in a judgmental way. Psychological flexibility has been positively correlated with perceived quality of life and affective well-being (4, 5), and may be particularly relevant for clinicians as it appears to be related to behaviors such as performance, prejudice, and the ability to learn new things (6). In fact, an intervention based on the theoretical construct of psychological flexibility was shown to significantly improve burnout and stigmatizing attitudes in providers counseling substance-abusing clients (7).

Several instruments have been developed to measure psychological flexibility in clinical and college samples. Although the construct appears relevant to the training and development of health care providers, research has not yet evaluated measures of psychological flexibility in medical learners. Therefore, our project sought to evaluate the validity of responses to three measures of psychological flexibility in a medical education sample. We gathered evidence related to five aspects of construct validity, as described by Downing (8): content, response process, internal structure, relationship with other variables, and consequences.

## Methods

We invited fourth-year medical students and first- and second-year residents (*N*=660) at our institution to complete an online, cross-sectional survey in the spring and summer of 2011. We selected this population because, although still in their medical training, subjects had already experienced a significant volume of clinical encounters. The survey contained a consent screen with all of the required elements of brief informed consent. Participation was voluntary and the study was approved by our Human Assurance Committee (IRB). Students and residents were informed that the survey was not a performance evaluation and would not affect their academic or work standing in any way. Participants were entered in a draw for 1 of 18 iPod shuffles.

### Measures of psychological flexibility

Theoretically, psychological flexibility is established through six key processes:

1. *Acceptance*: the active embrace of internal experiences (e.g., feeling rather than fighting anxiety);
2. *Cognitive defusion*: the capacity to decrease the believability of, or attachment to, internal thoughts and experiences;
3. *Being present*: present moment awareness and non-judgmental contact with experiences;
4. *Self as context*: the ability to observe and experience oneself as not defined by one's beliefs, emotions, and experiences but instead to see oneself as the context, or location, in which these experiences occur;
5. *Values*: being consistent with one's values rather than trying to avoid or comply with a different set of values; and
6. *Committed action*: developing effective actions linked to chosen values (5).

Based upon this six-process conceptual model, we selected three measures designed to assess psychological flexibility:

1. The Acceptance and Action Questionnaire-II (AAQ-II) is a seven-item, holistic measure of psychological flexibility, with items targeted to several of the six key processes: defusion, acceptance, and committed action (example item: ‘I worry about not being able to control my worries and feelings’.) (5, 9). Each item is followed by a seven-category response scale, ranging from 1 ‘Never true’ to 7 ‘Always true’. Higher scores indicate greater psychological inflexibility (total score range: 7–49, calculated as the sum of the item responses). Previous research has reported a mean Cronbach's *α* of 0.84 and mean test–retest reliabilities of 0.81 and 0.79 at 3 and 12 months, respectively (9). Factor analyses have established a unidimensional structure; and higher scores on the AAQ-II, indicative of greater psychological inflexibility, have been significantly correlated with psychological distress, absenteeism, and thought suppression (9).
2. The Cognitive Fusion Questionnaire-28 (CFQ-28) is designed to elicit one's level of cognitive fusion (example item: ‘I find it easy to view my thoughts from a different perspective’.) (10, 11). Respondents rate each of 28 items on the same seven-category response scale used with the AAQ-II. Higher scores on the CFQ indicate greater cognitive fusion (total score range: 28–196, calculated as a sum of the responses). Responses on the CFQ-28 are correlated in theoretically predicted directions with other measures of psychological flexibility, mindfulness, life satisfaction, and thought control. Studies demonstrate satisfactory reliability (Cronbach's *α*=0.86) and a theoretically consistent two-factor structure (i.e., fusion and defusion) (10, 11).
3. The Mindful Attention and Awareness Scale (MAAS) is a 15-item instrument designed to measure present moment attention and awareness (example item: It seems I am “running on automatic”, without much awareness of what I'm doing.) (12). The MAAS total score is derived by obtaining the mean score for the 15 items, each scored on a six-category response scale (1 ‘Almost always’ to 6 ‘Almost never’), with higher scores indicating greater mindfulness (13). Data indicate that the MAAS is a single-factor measure with adequate internal consistency of responses (Cronbach's *α*=0.82–0.87) and evidence to support convergent and discriminant validity (13).

#### Evaluation of validity

We gathered evidence related to five aspects of construct validity, as described by Downing: content, response process, internal structure, relationship with other variables, and consequences (8). All statistical analyses were performed using SAS 9.2. Statistical significance was assessed using an alpha level of 0.05.

### Content evidence

A team of 11 investigators, including two psychologists with particular expertise in psychological flexibility, identified measures of psychological flexibility from the literature. Measures were vetted for content related to the six key processes of psychological flexibility, ease of administration, prior validity evidence, and translation to a medical education sample. As psychological flexibility is a multi-faceted construct, we elected to study three measures in this project. The AAQ-II was selected for its unifactorial capture of psychological flexibility (9). The CFQ-28 and MAAS were selected for their ability to capture individual processes associated with psychological flexibility, namely cognitive fusion/defusion and mindfulness (10, 11, 13).

### Response process

We performed analyses to evaluate response patterns by determining the number of missing values for each scale per observation (Table 1) and evaluating for patterns of missing data. If an individual omitted responses to >20% of the items for each scale, his/her responses were excluded from further analysis. To allow for observations with missing items (for those with ≤20% missing), the average of the non-missing items was used to replace the missing values. We chose this technique because person-mean imputation has been shown to provide good estimates when ≤20% of responses are missing (14, 15). To minimize error with test administration, we administered the survey through One45, an online evaluation system used at our institution, with which all medical students and some residents are already familiar.

**Table 1:** Missing items for each of the three psychological flexibility scales

Number of items missing	Acceptance and Action Questionnaire-II (out of 7) *n* (%) *N*=238	Mindful Attention and Awareness Scale (out of 15) *n* (%) *N*=238	Cognitive Fusion Questionnaire-28 (out of 28) *n* (%) *N*=238
0	234 (98)	227 (95)	208 (87)
1	3 (1)	8 (3)	19 (8)
4			
5	1 (0)		
6			1 (0)
7			
9			1 (0)
11			1 (0)
15		3 (1)	
22			1 (0)
24			1 (0)
28			6 (3)

### Internal structure

The Shapiro–Wilk test was used to assess normality of the scales, and inter-item reliability was determined using Cronbach's α. We performed Confirmatory Factor Analyses (CFA) for the three psychological flexibility measures, using unweighted least-squares estimation due to the ordinal and non-normal nature of the data, as fit indices may be underestimated with maximum likelihood estimation in this circumstance (16). We evaluated several indices to determine the fit of the CFA with the hypothesized factor structure for each measure: (1) Standardized Root Mean-Square Residual (SRMR); (2) Goodness-of-Fit Index (GFI); (3) Adjusted Goodness-of-Fit Index (AGFI); (4) Bentler Comparative Fit Index (CFI); and (5) Bentler–Bonett Non-Normed Fit Index (NNFI). Literature suggests that the SRMR should be ≥0.05 for a good fit, with values ≥0.10 interpreted as acceptable (17). Values of ≥0.95 and ≥0.90 indicate a good fit for the GFI and AGFI, respectively (17). CFI and NNFI values ≥0.95 can be interpreted as an acceptable fit (17).

### Relationship with other variables

**Fig. 1:**
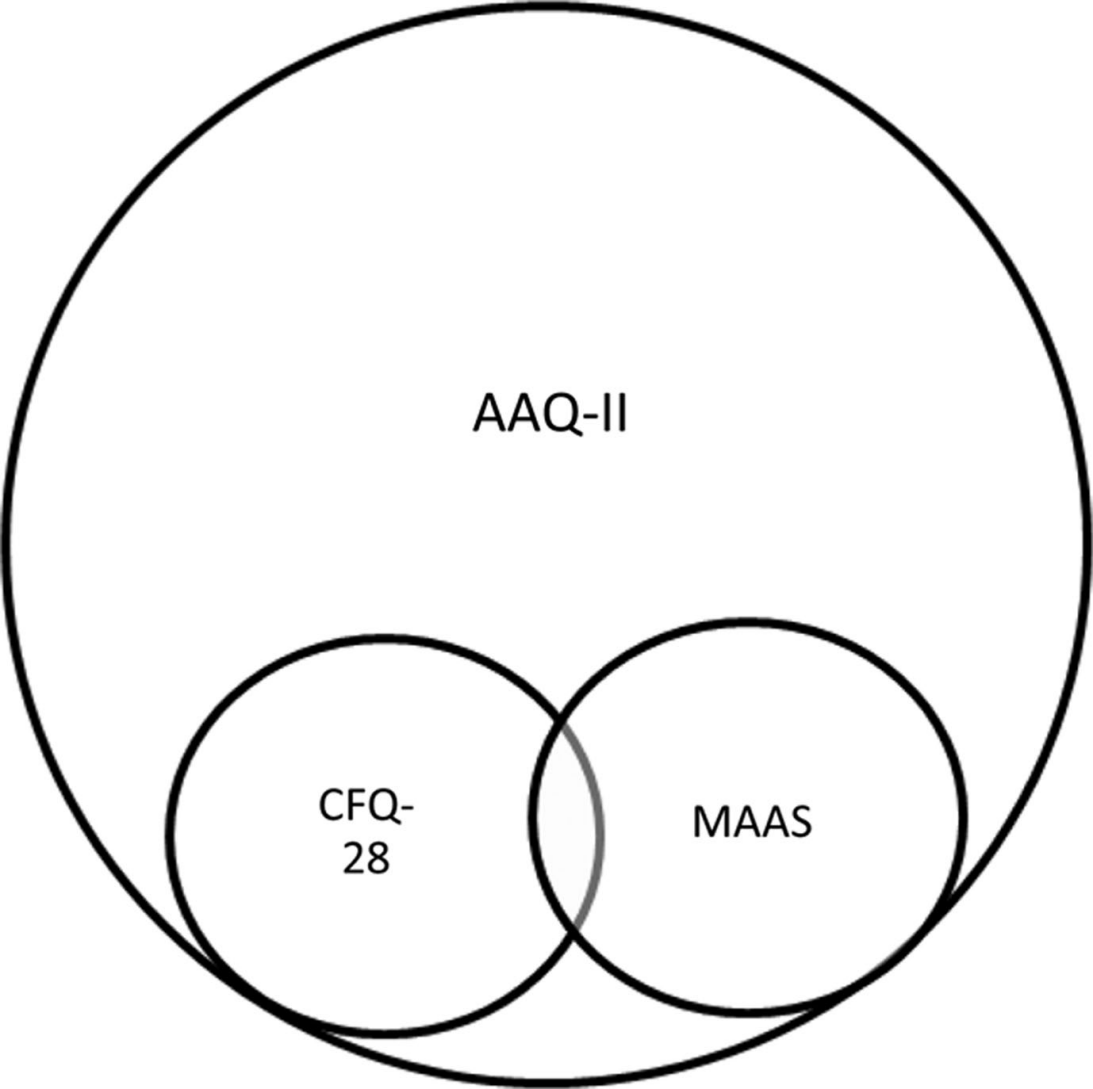
Theoretical relationship among the AAQ-II, CFQ-28, and MAAS.

We evaluated relationships with other variables in two ways: (1) correlations of scores on the three measures of psychological flexibility with one another and (2) correlations between scores on the three measures of psychological flexibility and scores on the Satisfaction With Life Scale (SWLS) (18). Given that the AAQ is a holistic measure of psychological flexibility, and that the CFQ-28 and MAAS capture related but not identical processes involved in psychological flexibility (Fig. 1), we hypothesized that scores on these measures would be moderately correlated with one another.

Previous research has demonstrated a significant, moderate correlation between psychological flexibility and psychological well-being. Thus, we hypothesized that scores on the AAQ, CFQ-28, and MAAS would be significantly correlated with life satisfaction, a component of psychological well-being. We administered the SWLS, a five-item measure of life satisfaction, as part of our survey (range: 5–35). Studies have reported good inter-item reliabilities using the scale and convergent validity with other measures of well-being (18).

As a secondary test of relationships among variables, we examined the correlation of scores on our three measures of psychological flexibility with scores on a measure of empathy. Given that people who demonstrate higher psychological flexibility are more comfortable with their own emotional distress (5), we hypothesized that they would be more empathic toward others because they would be more willing to experience others’ distress. To evaluate this, we included the Interpersonal Reactivity Index (IRI), a 28-item measure of four facets of empathy: perspective taking (IRI-PT; i.e., ability to adopt another's point of view); empathic concern (IRI-EC; i.e., compassion toward unfortunate others); personal distress (IRI-PD; i.e., discomfort when witnessing others in harm); and fantasy (IRI-FS; i.e., identification with fictitious characters) (19). Research has shown inter-item reliabilities >0.7 for each of the scales and test–retest reliabilities >0.6 (19). The IRI has been used in multiple medical education and physician samples (20, 21), with inter-item reliabilities of 0.72–0.77 on the scales and scores correlated with other measures of empathy in theoretically predicted directions (20, 21).

Finally, given items on the three measures of psychological flexibility such as ‘It is OK to have inconsistent thoughts on the same subject’ (CFQ) and ‘I rush through activities without being really attentive to them’ (MAAS), we anticipated that students may be tempted to provide a socially desirable response to some items. Therefore, we included the Medical Social Desirability Scale as a control variable (22). The Medical Social Desirability Scale is a seven-item, true-false measure developed to assess social desirability in a medical sample (range: 7–14) (22). Scores on the measure have demonstrated questionable reliability (*α*=0.62) but are specific to medical care and have been significantly correlated with scores on a generic measure of social desirability (22).

We calculated Pearson product–moment correlations, and Spearman's rho correlations where appropriate, to determine the relationship among scores on the three psychological flexibility scales and the relationship between scores on each of the three psychological flexibility scales with: the SWLS, the four subscales of the IRI, and the Medical Social Desirability Scale.

### Consequences

To evaluate evidence of consequential validity, we examined the effect of score cutoffs on the AAQ-II, as suggested by the literature. Bond et al. defined an AAQ-II score above a range of 24–28 as a preliminary cutoff to indicate clinically relevant distress (9). We used descriptive statistics to report the percentage of our sample scoring ≥24 and 28, respectively, and used the Wilcoxon–Mann–Whitney test to compare life satisfaction and empathy between learners scoring below and above these two cutoffs. We anticipated that respondents scoring above the clinically relevant cutoffs (i.e., clinically relevant psychological inflexibility) would score lower on life satisfaction and empathy.

## Results

### Response process

Two hundred and seventy-five students and residents responded to the survey. The majority of respondents were fourth-year medical students (*n*=176; 64%). Thirty-seven participants did not respond to any questions other than demographics, and were therefore dropped from all analyses (final completed surveys=36% of invited sample). Excluding these 37, at least 95% of respondents answered over 80% of the items on each scale (Table 1). We did not find any patterns of missing data within the responses. Response rates were highest among students at the beginning of the fourth year (response rate =44%) and residents at the end of their internship or second year (response rate =43%).

### Internal structure

Responses on the AAQ-II, CFQ-28, and MAAS demonstrated excellent internal consistency (Table 2). The goodness-of-fit indices from the CFA indicated that the hypothesized factor structures for all three scales (AAQ-II: one-factor; CFQ-28: two-factor; and MAAS: one-factor) (9–11, 13) were supported.

**Table 2:** Inter-item reliabilities and goodness-of-fit indices for the AAQ-II, MAAS, and CFQ-28

	Acceptance and Action Questionnaire-II	Mindful Attention and Awareness Scale	Cognitive Fusion Questionnaire-28
Mean (SD)	14.81 (7.10)	4.32 (0.80)	87.84 (19.46)
Range	7–43	2.47–6.0	39–142
Cronbach's *α*	0.93	0.92	0.95
Goodness-of-Fit Indices			
SRMR	0.05*	0.07	0.09
GFI	0.99	0.98	0.97
AGFI	0.99	0.97	0.96
CFI	1.0†	0.98	0.96
NNFI	0.99	0.98	0.96

* SRMR=0.048 for the AAQ-II

† CFI=0.996 for the AAQ-II.

Responses to all IRI scales demonstrated acceptable to good internal consistency (IRI-EC: *α*=0.81; IRI-PT: *α*=0.79; IRI-FS: *α*= 0.81; and IRI-PD: *α*=0.78). Data for the Medical Social Desirability Scale demonstrated a Cronbach's *α* of 0.65. Item analyses demonstrated that removal of items from the scale would not increase the Cronbach's *α*. There was little variability in scores on the Medical Social Desirability Scale, with 56% of respondents scoring a maximum score of 14 and 87% scoring ≥12.

### Relationship to other variables

Scores on the three psychological flexibility scales were statistically significantly correlated with one another and were also significantly correlated with scores on the SWLS, with greater psychological flexibility related to higher life satisfaction (Table 3). Psychological flexibility scores on all three measures were statistically significantly correlated with scores on the IRI-PD but not with the IRI-EC or IRI-PT scores.

**Table 3:** Correlations between the Acceptance and Action Questionnaire-II (AAQ-II), Mindful Attention and Awareness Scale (MAAS), Cognitive FusionQuestionnaire-28 (CFQ-28), and other measures

	AAQ	MAAS	CFQ	SWLS	IRI-PT	IRI-FS	IRI-PD	IRI-EC	SD
AAQ	–								
MAAS	−0.53*	–							
CFQ	0.63*	−0.45*	–						
SWLS	−0.40*	0.28*	−0.47*	–					
IRI-PT	−0.04	−0.03	−0.09	0.19*	–				
IRI-FS	0.21*	−0.13	0.07	0.06	0.27*	–			
IRI-PD	0.33*	−0.23*	0.53*	−0.32*	−0.32*	−0.03	–		
IRI-EC	0.05	0.08	−0.13	0.19*	0.57*	0.47*	−0.21*	–	
SD	−0.12	0.12	−0.02	−0.04	0.03	0.02	−0.02	0.15*	–

Other measures: Satisfaction With Life Scale (SWLS); Interpersonal Reactivity Index-Perspective Taking (IRI-PT); Interpersonal Reactivity Index-Fantasy Scale (IRI-FS); Interpersonal Reactivity Index-Personal Distress (IRI-PD); Interpersonal Reactivity Index-Empathic Concern (IRI-EC); Medical Social Desirability Scale (SD).

* *P*<0.05.

### Consequences

Twenty-nine respondents (12.2%) scored ≥24 on the AAQ-II, and 18 (7.6%) scored ≥28 (Table 4). Life satisfaction was significantly lower and PD was significantly higher in respondents scoring at or above either cutoff. IRI-EC and PT scores were lower in learners scoring ≥28, but this did not reach statistical significance.

**Table 4:** Life satisfaction and empathy by level of psychological flexibility

	Acceptance and Action Questionnaire-II <24	Acceptance and Action Questionnaire-II ≥24	*P*	Acceptance and Action Questionnaire-II <28	Acceptance and Action Questionnaire-II ≥28	*P*
SWLS, mean (SD)	27.52 (5.36)	21.33 (5.64)^1^	<0.01	27.28 (5.50)	20.19 (4.90)^2^	<0.01
IRI-EC, mean (SD)	26.13 (4.75)	25.19 (4.28)	0.25	26.15 (4.69)	24.25 (4.64)	0.08
IRI-PT, mean (SD)	24.43 (4.71)	24.23 (4.79)	0.46	24.52 (4.75)	22.80 (3.90)	0.07
IRI-FS, mean (SD)	22.48 (5.59)	24.37 (4.83)	0.13	22.72 (5.69)	22.50 (2.68)	0.89
IRI-PD, mean (SD)	15.56 (4.40)	19.62 (4.06)^3^	<0.01	15.65 (4.39)	21.00 (3.43)^4^	<0.01

1 Cohen's *d*=1.12

2 Cohen's *d*=1.36

3 Cohen's *d*=0.96

4 Cohen's *d*=1.36.

## Discussion

This study extends the literature on the assessment of self-awareness in medical education. Our innovation is in testing these measures, and thereby examining the construct of psychological flexibility, in a sample of medical students and residents. Our findings from responses to the AAQ-II, CFQ, and MAAS provide preliminary evidence of construct validity in our sample, particularly related to internal structure of these measures.

Our results demonstrated good internal consistency among responses to the AAQ-II, CFQ-28, and MAAS. Measures of inter-item reliability were satisfactory and consistent with values in other samples (9, 11, 13). The high Cronbach's *α* on the CFQ-28 in our sample may even indicate some item redundancy. A shorter 13-item version of the CFQ is in development although, at the time of this study, the 28-item version was recommended by the developers (email conversation with D Gillanders, Deputy Director, Clinical Psychology Programme, University of Edinburgh, November 17, 2010). Our CFA also supported the previously reported factor structures for all three measures, and our findings demonstrated expected correlations of the AAQ-II, MAAS, and CFQ with one another and with life satisfaction.

Respondents to our survey scored as more psychologically flexible than other samples in the literature. For example, our mean AAQ-II score was lower (i.e., indicating more psychological flexibility) than scores in samples of undergraduate students and bank employees (9). Similarly, our mean of 85.1 on the CFQ-28 was lower than a mean of 97.9 among undergraduate and postgraduate students (10). Our sample also scored higher on the MAAS (i.e., more mindful) than samples of psychology students, clinical patients, and adolescents (13, 23). However, most of the scores in other samples were within one standard deviation of our mean score. Still, medical students and residents may represent a more psychologically flexible sample, particularly given that they have been selected over time though a rigorous process, which perhaps might weed out more psychologically inflexible individuals. Higher scores on psychological flexibility may also represent a unique characteristic among our sample of respondents. However, the very elevated social desirability scores of this sample (i.e., 56% scored the maximum on the measure) suggest that the psychological flexibility scores may be high because respondents were attempting to present themselves in the most positive light. This would not be revealed in the correlations between the psychological flexibility scores and the medical social desirability scores because there was so little variability in scores on the social desirability variable. Future research should examine in more detail whether psychological flexibility differs between health care trainees, other professional students, and the general population.

In our sample, psychological flexibility was significantly correlated with life satisfaction at a small-medium effect size (absolute value of *r*=0.28–0.47, Table 3), consistent with our expectations from the literature (5). In addition, being less psychologically flexible was significantly associated with PD when seeing others in harm. The association between psychological flexibility and PD represented a small-large effective size, depending on the psychological flexibility measure. Such an association could potentially impact learners’ well-being, burnout, and interactions with patients and the health care team.

We did not find a significant correlation between psychological flexibility and EC, as hypothesized. Several explanations should be considered. First, the IRI looks at empathy, but not specifically empathy in a medical context. The presence of a correlation between psychological flexibility and PD, but the lack of correlation between psychological flexibility and EC may also represent a ‘me’ vs. ‘you’ perspective. In our sample, psychological flexibility was significantly correlated with scores on the IRI-PD, which captures *personal discomfort and anxiety* when witnessing others undergoing negative experiences. Yet, it was not significantly correlated with the IRI-EC, which captures experiencing *compassion toward others*, or the IRI-PT, which captures the ability to adopt *others’ points of view*. Finally, the lack of correlation between psychological flexibility and EC in our sample may reflect that these theoretical constructs are not overlapping. Other research has failed to demonstrate a significant correlation between mindfulness and EC and has found inconsistent correlations between mindfulness and PT (24) and between the AAQ-II and EC (personal communication between A House and R Viladarga, General Adult Psychology, Washington University, November 13, 2011). Yet, in general, little literature has focused on the relationship between psychological flexibility and empathy. In addition, as interventions targeting psychological flexibility continue to be considered as potential mechanisms for impacting empathic skills (25) and have demonstrated impact on providers’ attitudes toward clients (7), this is an important area for further study.

This study has several limitations, including a sample from one institution. Our response rate was limited, likely affected by: (1) survey timing (near graduation); (2) hectic clinical schedules; and (3) length of the survey (over 100 items). However, response rates among beginning of fourth-year medical students and first- and second-year residents were comparable to physician response rates on other national surveys. While a low response rate can lead to selection bias, our study's intent was not to generalize the amount of psychological flexibility to other samples but rather to examine the potential evidence of construct validity among our sample of respondents. The anonymous nature of our survey did not permit us to compare demographics between respondents and non-respondents. While we did measure response rates and patterns of missing data, we did not evaluate respondents’ comprehension of survey items, which is an element of response process. Finally, the CFQ-28 and AAQ-II are relatively new measures. Responses should be reexamined as research continues to evolve, allowing for further refinement of these instruments and the constructs they measure.

Limitations notwithstanding, our study demonstrates preliminary evidence of construct validity related to the assessment of psychological flexibility in a medical education sample. Also, our findings suggest a potentially important relationship between psychological flexibility and PD. This finding may open a pathway for identifying at-risk trainees. Future research should continue to explore the degree of consequential validity related to measuring psychological flexibility in samples of medical trainees. In particular, our future research on this topic will evaluate the following: potential relationships between psychological flexibility, PD, coping abilities, and patient care; refining measures of psychological flexibility for use in medical education samples; potential impact of social desirability on measuring these constructs; collaborations with samples at other institutions; and potential use of interventions targeting psychological flexibility in medical students and residents.

## Previous presentations

The abstract of an earlier version of this article was presented as a poster at the 2012 Southern Group on Educational Affairs Meeting.
